# Distinct and overlapping roles of STAG1 and STAG2 in cohesin localization and gene expression in embryonic stem cells

**DOI:** 10.1186/s13072-020-00353-9

**Published:** 2020-08-10

**Authors:** Nicole L. Arruda, Zachary M. Carico, Megan Justice, Ying Frances Liu, Junjie Zhou, Holden C. Stefan, Jill M. Dowen

**Affiliations:** 1 Curriculum in Genetics and Molecular Biology, University of North Carolina at Chapel Hill, Chapel Hill, NC 27599 USA; 2 Cancer Epigenetics Training Program, University of North Carolina at Chapel Hill, Chapel Hill, NC 27599 USA; 3 Integrative Program for Biological and Genome Sciences, University of North Carolina at Chapel Hill, Chapel Hill, NC 27599 USA; 4 Department of Biochemistry and Biophysics, University of North Carolina at Chapel Hill, Chapel Hill, NC 27599 USA; 5 Department of Biology, University of North Carolina at Chapel Hill, Chapel Hill, NC 27599 USA; 6 Lineberger Comprehensive Cancer Center, University of North Carolina at Chapel Hill, Chapel Hill, NC 27599 USA

**Keywords:** Cohesin, STAG, Transcription, Gene expression, Enhancers, Promoters, Stem cell, CTCF, CRISPR/Cas9

## Abstract

**Background:**

The three-dimensional organization of the genome in the nucleus plays an integral role in many biological processes, including gene expression. The genome is folded into DNA loops that bring together distal regulatory elements and genes. Cohesin, a ring-shaped protein complex, is a major player in the formation of DNA loops. Cohesin is composed of a core trimer and one of two variant STAG subunits, STAG1 or STAG2. It is not understood whether variant STAG proteins give rise to cohesin complexes with distinct functions. Recent studies have begun to characterize the roles of STAG1 and STAG2, with partially contradictory results.

**Results:**

Here, we generate stable single-knockout embryonic stem cell lines to investigate the individual contributions of STAG1 and STAG2 in regulating cohesin chromosomal localization and function. We report both overlapping roles for STAG1 and STAG2 in cohesin localization and somewhat distinct roles in gene expression. STAG1 and STAG2 occupy the same sites across the genome, yet do not exist together in a higher order complex. Despite their shared localization, STAG1 and STAG2 have both distinct and redundant effects on gene expression. Loss of both STAG1 and STAG2 causes widespread transcriptome dysregulation, altered cohesin DNA occupancy, and reduced cell proliferation.

**Conclusions:**

Together, this work reveals the requirement of at least one STAG protein for proper cohesin function. STAG1 and STAG2 have independent roles in cohesin localization and both overlapping and distinct roles in gene expression. The roles of STAG1 and STAG2 in mouse embryonic stem cells may be somewhat different than in other cell types, due to their relative expression levels. These results advance our understanding of the link between mammalian genome organization and gene expression during development and disease contexts.

## Background

The spatial organization of the eukaryotic genome is fundamentally important for proper gene expression. Cohesin and CTCF play essential roles in establishing and regulating three-dimensional genome structure [1–4]. Cohesin functions in sister chromatid cohesion, DNA repair, replication, and gene expression, but the molecular mechanisms that underlie such diverse roles remain poorly understood [5–7]. It is hypothesized that accessory subunits, like STAG1 and STAG2, could alter the properties and functions of the core cohesin complex. Cohesin is a ring-shaped protein complex comprised of the core subunits SMC1A, SMC3, and RAD21. This core complex can associate with either STAG1 or STAG2 [8, 9] along with several other regulatory proteins. Importantly, STAG1 and STAG2 are not thought to function outside of the cohesin complex.

Proper cohesin function is required for normal development and cellular function. Homozygous loss of core cohesin ring components is lethal to cells [10, 11], however mutations in cohesin subunits and cohesin regulators can be tolerated and are observed in various cancers including myeloid leukemia, glioblastoma, Ewing sarcoma, and melanoma [12–14]. The STAG proteins are frequently mutated in cancers, but it is not clear if such defects contribute to disease. STAG proteins also appear to be necessary for normal development, with STAG1 knockout mice showing developmental defects and embryonic lethality [15, 16]. Loss of STAG proteins is predicted to cause misexpression of genes, thereby leading to disease. Whereas acute loss of cohesin causes minimal transcriptional changes, it is likely that the duration of STAG loss is important for widespread gene expression defects [10, 11, 17]. A deeper understanding of how cohesin subunits impact transcriptional regulation is needed to elucidate the molecular mechanisms by which cohesin controls gene expression and to reveal how defects in these functions may contribute to disease.

Defining the individual roles of STAG1 and STAG2 will provide insight into how variant cohesin complexes exhibit different functions in cellular processes and disease. The two STAG proteins display 75% conservation at the amino acid level, with the N-terminal and C-terminal regions showing the most divergence [8]. Both proteins contribute to sister chromatid cohesion on chromosome arms, but cohesin-STAG1 is specifically required for cohesion at telomeres, and cohesin-STAG2 for cohesion at centromeres [15, 18, 19]. Cohesin-STAG1 is also implicated in DNA replication at telomeres, because its loss results in decreased telomeric replication and subsequent aneuploidy [15]. Cohesin-STAG2 participates in repair of DNA damage and promotes replication fork progression [20–22].

Recent work has implicated both STAG1 and STAG2 in regulating gene expression and genome organization, however, the molecular basis of STAG-dependent gene regulation has been controversial. Although some studies suggest STAG1 and STAG2 localize to many shared sites across the genome, other studies identify and highlight smaller classes of distinct sites with possibly different functions in various mammalian cell types [23–27]. Some reports have observed that cohesin-STAG1 localizes to CTCF sites and TAD boundaries, whereas cohesin-STAG2 is found at enhancers, promoters, and Polycomb Domains [23, 24]. However, a contradictory report found that STAG1, but not STAG2, is enriched upstream of transcription start sites [16]. In general, acute depletion of STAG1 or STAG2 is thought to cause selective transcriptional defects, suggesting that the two proteins have at least partially distinct roles in gene control [23–26]. STAG1 and STAG2 might differentially contribute to higher order genome organization, with STAG1 appearing to mediate more long-range interactions and STAG2 mediating more mid-range interactions [23–25, 27]. These distinct types of DNA loops could be due to differential stability of cohesin rings on DNA. Cohesin-STAG1 interacts more strongly with CTCF than cohesin-STAG2, and cohesin-STAG1 is more resistant to removal from DNA by the cohesin unloading factor WAPL than cohesin-STAG2 [27]. Consistent with this idea, cohesin-STAG2 rings associate more strongly with WAPL than cohesin-STAG1 [23, 28]. Deeper knowledge of STAG protein function is important for understanding how variant cohesin complexes might differentially regulate gene expression and three-dimensional genome organization.

Here we show that cohesin-STAG1 and cohesin-STAG2 have nearly identical localization patterns across the embryonic stem cell genome. Stable loss of STAG1 or STAG2, following CRISPR/Cas9 genome editing, has minimal effects on the distribution and levels of cohesin on the genome. Nevertheless, gene expression analysis in isogenic *Stag1*^−*/*−^ or *Stag2*^−*/*−^ mESCs reveals only partially overlapping roles for these two proteins in transcriptional regulation. Depletion of STAG1 in *Stag2*^−*/*−^ cells reveals their redundant functions in cohesin occupancy on the genome, cell proliferation, and gene expression at a large set of genes not sensitive to the loss of either individual STAG protein.

## Results

### Overlapping distribution of cohesin-STAG1 and cohesin-STAG2 on the genome

**Fig. 1:**
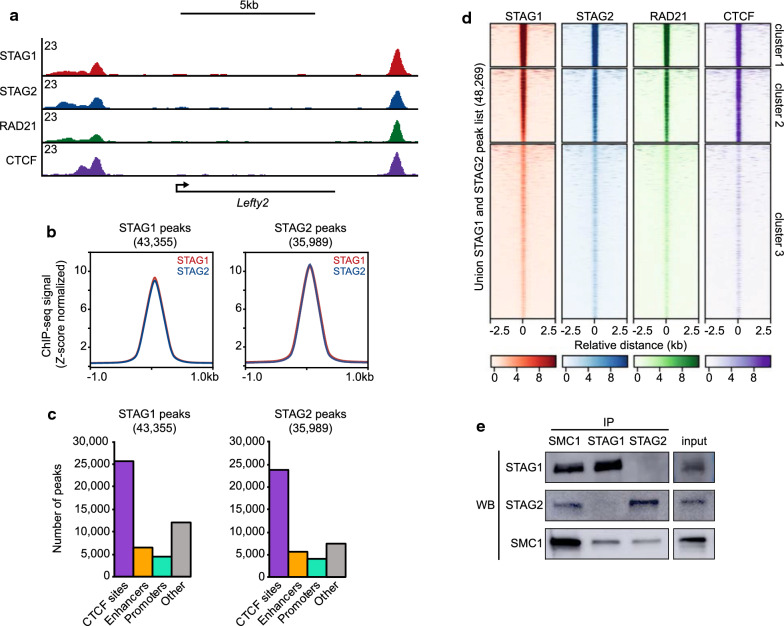
Overlapping distribution of cohesin-STAG1 and cohesin-STAG2 on the genome. **a** Genome browser tracks showing ChIP-seq signal for STAG1, STAG2, RAD21, and CTCF (*Z*-score normalized). **b** Average signal plots of STAG1 and STAG2 signal at STAG1 peaks and STAG2 peaks (*Z*-score normalized). **c** Frequency of peaks overlapping known functional elements in the genome: CTCF sites, enhancers, promoters, or other (none of the above). **d** Clustered heatmaps displaying STAG1, STAG2, RAD21, and CTCF signal (*Z*-score normalized) at a union list of STAG1 and STAG2 peaks. **e** Western blot analysis following co-immunoprecipitation of SMC1, STAG1, and STAG2 from nuclear lysates

To investigate the localization of STAG1 and STAG2 in mouse embryonic stem cells (mESCs), we performed chromatin immunoprecipitation followed by high-throughput sequencing (ChIP-seq) (Additional file 1: Table S1). In order to quantitatively compare signals between samples, a spike-in of human chromatin was added prior to the immunoprecipitation and used to normalize the datasets. Peak calling with MACS2 [29] (FDR < 0.01) identified 43,355 STAG1 peaks and 35,989 STAG2 peaks in the mESC genome. ChIP-seq for RAD21 and CTCF was also performed and revealed that STAG proteins display a strong overlap with the core cohesin complex and CTCF (Fig. 1a, Additional file 2: Figure S1A). Strikingly, STAG1 and STAG2 show very similar ChIP-seq signals across the genome. At both STAG1 peaks and STAG2 peaks, there are similar levels of both STAG proteins (Fig. 1b). While previous studies have shown that STAG1 preferentially occupies CTCF sites and STAG2 preferentially occupies enhancers and promoters [23, 24], we observe no preference for STAG1 or STAG2 peaks overlapping CTCF sites, enhancers, or promoters (Fig. 1c). RAD21 peaks show a similar distribution to STAG1 and STAG2 across these functional elements (Additional file 2: Figure S1B). Importantly, our STAG1 and STAG2 ChIP-seq datasets show strong overlap with previously reported STAG1 and STAG2 ChIP-seq datasets in mESCs that were generated with different antibodies (Pearson correlation, *R*^2^ = 0.95 and 0.80 for STAG1 and STAG2, respectively) [23] (Additional file 2: Figure S1C). Notably, use of a higher false discovery rate (FDR < 0.05) does not greatly impact the number of peaks called or alter the strong overlap of the two datasets (Additional file 2: Figure S1D). Clustered ChIP-seq signal at a union list of 48,269 STAG1 and STAG2 peaks shows that STAG1 levels and STAG2 levels are correlated, and fails to reveal sites in which a single STAG protein is preferred over the other (Fig. 1d). Importantly, these ChIP-seq datasets are of high quality with strong enrichment over background and a high proportion of reads are contained within peaks (Additional file 2: Figure S1E). Together, these data indicate that cohesin-STAG1 and cohesin-STAG2 exhibit very similar patterns of localization across the mESC genome.

Various models of cohesin function have been proposed, some involving a single cohesin ring complex and others requiring two rings to bring a pair of distal sequences into physical proximity [30–33]. Because our ChIP-seq data suggest that STAG1 and STAG2 localize to the same sites across the genome, we next investigated whether they exist in a complex. Co-immunoprecipitation (coIP) experiments performed under native conditions revealed that immunoprecipitation (IP) of STAG1 was able to co-purify the core cohesin subunit SMC1A but not STAG2. Likewise, IP of STAG2 was able to co-purify SMC1A but not STAG1 (Fig. 1e). Importantly, the SMC1A–STAG interaction was not dependent on DNA or RNA, since the immunoprecipitation was performed in the presence of a nuclease. This result demonstrates that STAG1 and STAG2 proteins do not exist together in a higher-order complex, corroborating a previous study that used both microscopy and Re-ChIP to demonstrate that STAG1 and STAG2 do not colocalize to the same piece of chromatin at the same time [25]. Together with the overlapping pattern of STAG1 and STAG2 localization, our work argues that individual cells within a population have either a cohesin-STAG1 or cohesin-STAG2 complex present at a specific site in the genome at any given time.

### Loss of a STAG protein causes minimal redistribution of the other STAG protein

**Fig. 2:**
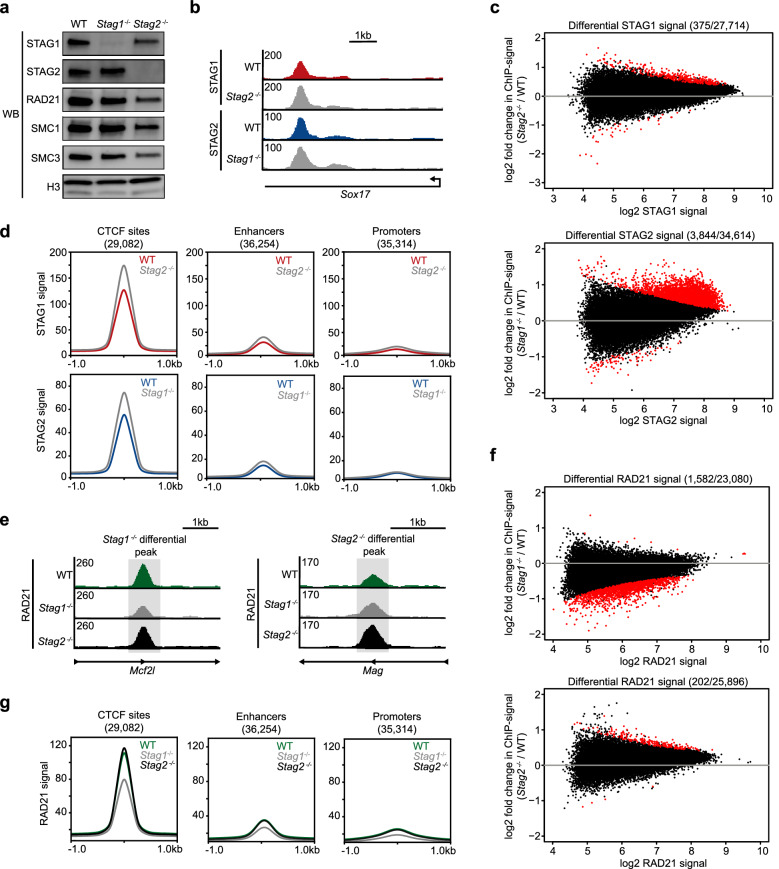
Cohesin distribution is minimally changed in *Stag1*^−*/*−^ and *Stag2*^−*/*−^ mESCs. **a** Western blot analysis of cohesin subunit levels in wild-type, *Stag1*^−*/*−^, and *Stag2*^−*/*−^ nuclear lysates. **b** Genome browser tracks showing STAG1 signal in wild-type and *Stag2*^−*/*−^ cells, and STAG2 signal in wild-type and *Stag1*^−*/*−^ cells. **c** MA plots showing differential enrichment of STAG1 signal between wild-type and *Stag2*^−*/*−^ cells. Differential enrichment of STAG2 signal between wild-type and *Stag1*^−*/*−^ cells is also shown. **d** Average signal plots of STAG1 signal at CTCF sites, enhancers, and promoters in wild-type and *Stag2*^−*/*−^ cells. STAG2 signal at CTCF sites, enhancers, and promoters in wild-type and *Stag1*^−*/*−^ cells is also shown. **e** Genome browser tracks showing RAD21 signal in wild-type, *Stag1*^−*/*−^, and *Stag2*^−*/*−^ cells at *Stag1*^−*/*−^ and *Stag2*^−*/*−^ differential peaks. **f** MA plots showing differential enrichment of RAD21 in *Stag1*^−*/*−^ and *Stag2*^−*/*−^ cells. **g** Average signal plots of RAD21 signal at CTCF sites, enhancers, and promoters in wild-type, *Stag1*^−*/*−^, and *Stag2*^−*/*−^ cells

We next evaluated whether the STAG proteins compensate for one another with regard to their occupancy of the genome. We used CRISPR/Cas9 genome editing to generate stable single-knockout mESC lines. Multiple clonal *Stag1*^−*/*−^ cell lines and *Stag2*^−*/*−^ cell lines were created and their homozygous deletions were confirmed by Sanger sequencing (Additional file 2: Figure S2A). Western blot analysis confirmed the loss of the target protein and demonstrated that the levels of other cohesin complex subunits were not strikingly changed (Fig. 2a). STAG1 ChIP-seq was performed in *Stag2*^−*/*−^ cells, employing a spike-in for quantification of relative signals. STAG1 occupancy on the genome was similar in wild-type and *Stag2*^−*/*−^ cells, with a high correlation between the two cell lines (Fig. 2b, Additional file 2: Figure S2B) and similar numbers of peaks identified (43,355 and 42,389, respectively). Most of the wild-type STAG1 peaks overlapped with STAG1 peaks in *Stag2*^−*/*−^ cells, and the non-overlapping peaks often showed signal for STAG1 in *Stag2*^−*/*−^ cells that fell short of the peak calling threshold (Additional file 2: Figure S2C). To identify sites of differential STAG1 enrichment, we used DiffBind [34]. Only 375 sites displayed differential STAG1 signal among a consensus list of 27,714 sites (1.35%) shared between wild-type and *Stag2*^−*/*−^ cells (Fig. 2c). Most of the 375 sites of differential STAG1 signal showed increased signal in *Stag2*^−*/*−^ cells relative to wild-type cells. STAG1 signal in both wild-type and *Stag2*^−*/*−^ cells frequently occupied CTCF sites and, to a lesser extent, enhancers and promoters (Fig. 2d).

STAG2 ChIP-seq in *Stag1*^−*/*−^ cells revealed moderate changes in STAG2 occupancy compared to wild-type cells (Fig. 2b, Additional file 2: Figure S2B). As we observed with our STAG1 analysis, STAG2 occupancy on the genome was similar in wild-type and *Stag1*^−*/*−^ cells, with similar numbers of peaks identified in each cell line (35,989 and 33,346, respectively) and most of the peaks in the two cell lines overlapped (Additional file 2: Figure S2C). Differential STAG2 signal was observed at 3844 sites from a consensus list of 34,614 STAG2 sites (11.1%) shared between wild-type and *Stag1*^−*/*−^ cells (Fig. 2c). The differential STAG2 signal was mostly increased in *Stag1*^−*/*−^ cells relative to wild-type cells. STAG2 signal in both wild-type and *Stag1*^−*/*−^ cells frequently occupied CTCF sites, and to a lesser extent, enhancers and promoters (Fig. 2d). Importantly, all STAG1 and STAG2 ChIP-seq datasets displayed similar enrichment profiles, supporting the quality of the samples, and antibody specificity was validated by ChIP-qPCR and western blot analysis (Fig. 2a, Additional file 2: Figure S2D–E). We conclude that, in the absence of one STAG protein, there is no major redistribution of the other STAG protein to new genomic sites. Furthermore, at the normally occupied STAG sites, there is not a large increase in the levels of remaining STAG protein, indicating that STAG proteins are limiting. We thus observe little evidence for STAG peaks that are specific to wild-type, *Stag1*^−*/*−^ cells, or *Stag2*^−*/*−^ cells. Additionally, fewer than 11% of peaks showed changes in STAG levels in wild-type, *Stag1*^−*/*−^ cells, or *Stag2*^−*/*−^ cells. Rather, many of the sites occupied by STAG proteins in wild-type cells are also occupied in *Stag1*^−*/*−^ cells or *Stag2*^−*/*−^ cells at similar levels.

### Cohesin localization is largely independent of STAG1 and STAG2

It has been suggested that the STAG proteins may serve as a protein–protein interface between CTCF and cohesin [35, 36]. We sought to investigate whether loss of a single STAG protein causes redistribution of the core cohesin complex across the genome, possibly by disruption of a cohesin–CTCF interaction. RAD21 ChIP-seq was performed with a spike-in control in wild-type, *Stag1*^−/−^, and *Stag2*^−*/*−^ cells (Fig. 2e). Similar numbers of cohesin peaks were identified in each cell line: 33,665 wild-type peaks, 26,920 peaks in *Stag1*^−*/*−^ cells, and 34,694 peaks in *Stag2*^−*/*−^ cells. RAD21 signal in either knockout cell line was positively correlated with the RAD21 signal in wild-type cells, and all datasets had similar ChIP-seq enrichment profiles (Additional file 2: Figure S2F–G). Most RAD21 peaks in the knockout lines overlapped wild-type RAD21 peaks, and the non-overlapping peaks often showed signal that fell short of the peak calling threshold (Additional file 2: Figure S2H). DiffBind was used to identify differential RAD21 signal among a consensus list of peaks, revealing 1582 RAD21 sites from a consensus of 23,080 sites (6.85%) that displayed differential signal in *Stag1*^−*/*−^ cells compared to wild-type. *Stag2*^−*/*−^ cells displayed only 202 sites from a consensus of 25,896 sites (0.78%) of differential RAD21 signal (Fig. 2f). RAD21 signal in wild-type, *Stag1*^−*/*−^, and *Stag2*^−*/*−^ cells frequently overlapped CTCF sites and, to a lesser extent, enhancers and promoters (Fig. 2g). Notably, these sites of decreased RAD21 signal in *Stag1*^−*/*−^ cells do not overlap sites of increased STAG2 signal in *Stag1*^−*/*−^ cells, revealing a small class of sites (1445) that lose the core cohesin ring, but do not gain cohesin-STAG2 (Additional file 2: Figure S2I). These results indicate that loss of a single STAG protein does not cause a major redistribution of the core cohesin complex to new, ectopic sites. Rather, at the population level, STAG1, STAG2, and RAD21 largely localize to the same sites across the genome and at similar levels in wild-type, *Stag1*^−*/*−^, and *Stag2*^−*/*−^ cells.

### Partially distinct and overlapping roles of STAG1 and STAG2 in gene expression

**Fig. 3:**
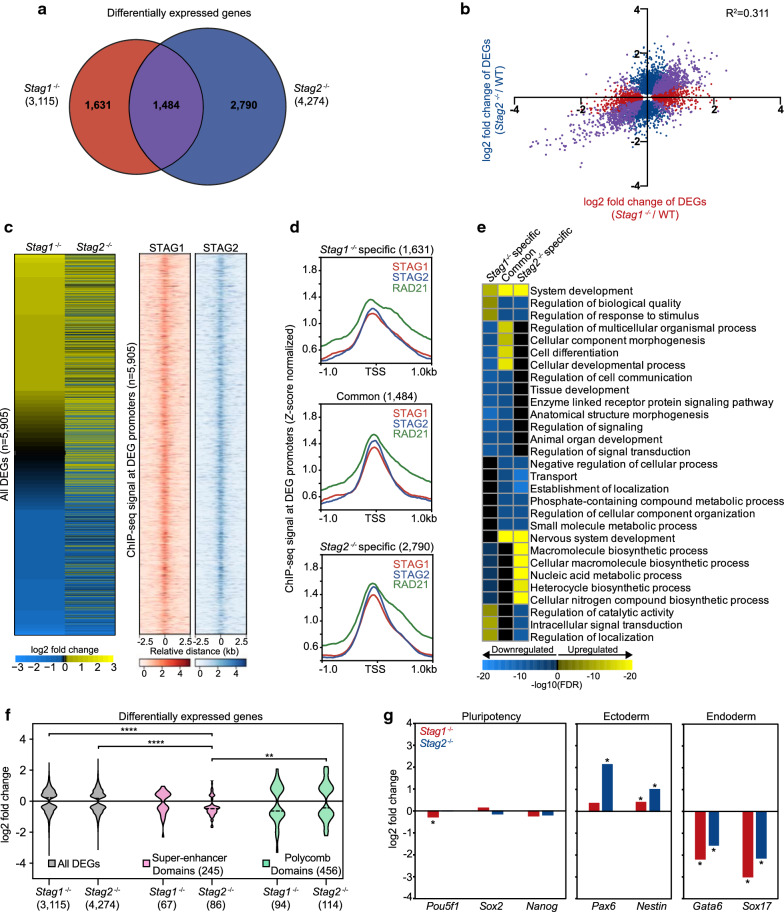
STAG1 and STAG2 display partially distinct and overlapping roles in gene expression. **a** Overlap of differentially expressed genes (DEGs) in *Stag1*^−*/*−^ cells compared to wild-type cells and *Stag2*^−*/*−^ cells compared to wild-type cells. Cells were treated with siGLO as a transfection control. **b** Correlation plot of log2 fold changes of DEGs. DEGs specific to *Stag1*^−*/*−^ are shown in red, DEGS specific to *Stag2*^−*/*−^ are shown in blue, and DEGs sensitive to the loss of either STAG (common) are shown in purple. **c** Heatmap of log2 fold changes for a combined list of DEGs in *Stag1*^−*/*−^ and *Stag2*^−*/*−^ cells. Heatmap of STAG1 and STAG2 ChIP-seq signal in wild-type cells at the promoters of the combined list of DEGs. **d** Average signal plots for STAG1, STAG2, and RAD21 ChIP-seq signal in wild-type cells at *Stag1*^−*/*−^ specific, common, and *Stag2*^−*/*−^ specific DEG promoters. **e** Gene Ontology (GO) terms for biological processes that are *Stag1*^−*/*−^ specific, *Stag2*^−*/*−^ specific, and common to both knockouts. **f** Violin plot depicting log2 fold changes for all DEGs, and those within Super-enhancer Domains and Polycomb Domains for *Stag1*^−*/*−^ and *Stag2*^−*/*−^ cells. Dotted lines indicate the mean. Asterisks indicate significant differences between groups (****p < 0.0001, **p < 0.01). **g** Bar graphs with log2 fold change of expression of cell identity genes, including those that represent pluripotency (*Pou5f1, Sox2, Nanog*), ectoderm (*Pax6* and *Nestin*), and endoderm lineages (*Gata6* and *Sox17*) in *Stag1*^−*/*−^ and *Stag2*^−*/*−^ cells. Asterisks indicate significant differences from wild-type cells (padj < 0.01)

To investigate the roles of STAG1 and STAG2 in gene regulation, we performed RNA-seq in wild-type, *Stag1*^−*/*−^, and *Stag2*^−*/*−^ cells. There were 3115 differentially expressed genes (DEGs) in *Stag1*^−*/*−^ cells and 4274 DEGs in *Stag2*^−*/*−^ cells compared to wild-type cells (padj < 0.1) (Additional file 3: Table S2). There were 1484 genes sensitive to the loss of either STAG protein, or commonly regulated (Fig. 3a). The majority of commonly regulated DEGs (1345 out of 1484) display gene expression changes in the same direction in both knockout cell lines and tend to have similar fold changes (Additional file 2: Figure S3A). However, overall the *Stag1*^−*/*−^ cells and *Stag2*^−/−^ cells have weakly correlated gene expression changes (*R*^2^ = 0.311) (Fig. 3b). Loss of a STAG protein caused similar numbers of up- and down-regulated genes (~ 1500 genes up and down for STAG1, and ~ 2100 genes up and down for STAG2) (Additional file 2: Figure S3B). Importantly, STAG1 and STAG2 transcript levels appear to be similar in wild-type mESCs and loss of either STAG protein did not significantly affect transcript or protein levels of the other STAG (Additional file 2: Figure S3C, Fig. 2a). This suggests that changes in gene expression are a consequence of loss of an individual STAG and not due to gain of function of the other STAG. We note that the *Stag1*^−*/*−^ replicate 1 cell line contains an in-frame deletion of 38 amino acids in the N-terminus of STAG1 that leads to normal Stag1 transcript levels but no detectable protein (Fig. 2a, Additional file 2: Figure S2A, S3C). Notably, the ratio of Stag2 transcripts to Stag1 transcripts in wild-type mESCs is 1.08, which is lower than most of the ~ 1000 cell lines in the Cancer Cell Line Encyclopedia [37], indicating that other cell types operate with a relatively higher level of cohesin-STAG2 than cohesin-STAG1 (Additional file 2: Figure S3D).

Despite the weak global correlation, many genes were similarly impacted by loss of either STAG protein, as observed by a similar trend of log2-fold changes at a merged list of 5905 DEGs (Fig. 3c). ChIP-seq signal for STAG1 and STAG2 in wild-type cells reveals that the promoters of DEGs tend to be occupied by STAG1 and/or STAG2. Importantly, there is not a major difference between STAG1, STAG2, and RAD21 wild-type ChIP-seq signal at *Stag1*^−*/*−^ specific, common, and *Stag2*^−*/*−^ specific DEG promoters (Fig. 3d). Comparing *Stag1*^−*/*−^ cells directly to *Stag2*^−*/*−^ cells revealed 1940 genes with higher transcript levels in *Stag1*^−*/*−^ than *Stag2*^−*/*−^ cells, and 1936 genes with higher transcript levels in *Stag2*^−*/*−^ than *Stag1*^−*/*−^ cells (Additional file 2: Figure S3E). These results indicate that STAG1 and STAG2 do not fully genocopy one another, as some genes are similarly impacted by loss of either STAG, while other genes are differentially impacted or not changed in one of the single-knockout cell lines. Gene ontology analysis of *Stag1*^−*/*−^ specific, common, and *Stag2*^−*/*−^ specific gene sets identified biological processes that were differentially impacted by loss of STAG1 or STAG2 (Fig. 3e, Additional file 4: Table S3). Biological processes tend to sort into those regulated by STAG1 or STAG2, with only one example (‘system development’) among the top 30 hits showing coordinated regulation by the two proteins, and several metabolic and biosynthetic pathways showing opposing regulation by the STAG proteins. It is surprising to find a small degree of overlap in the biological processes dysregulated by loss of STAG1 versus STAG2, given their similar occupancy patterns across the genome.

We next examined gene expression at insulated neighborhoods in *Stag1*^−*/*−^ and *Stag2*^−*/*−^ cells. Insulated neighborhoods are DNA loop structures formed by cohesin and CTCF, that control gene expression by restricting enhancer activity or spread of a chromatin state [38]. Two classes of insulated neighborhoods are Super-enhancer Domains and Polycomb Domains. Super-enhancer Domains (SDs) focus super-enhancer activity on genes inside of the DNA loop (Additional file 2: Figure S3F), and in cases of impaired loop function, genes inside the loop decrease in expression, while genes outside can increase in expression [38]. We hypothesized that loss of STAG1 or STAG2 could disrupt cohesin function at the anchors of insulated neighborhoods and impact the expression of genes in the local area. Indeed, both *Stag1*^−*/*−^ cells and *Stag2*^−*/*−^ cells displayed gene expression defects at individual SD examples, with target genes inside decreasing in expression and genes outside increasing in expression (Additional file 2: Figure S3G). However, when considering all DEGs contained within SDs, only *Stag2*^−*/*−^ cells displayed a significant decrease in expression relative to all DEGs (*p* < 0.0001) (Fig. 3f). Further analysis of the genes commonly or specifically impacted by loss of the STAG proteins showed that a high percentage of *Stag2*^−*/*−^ specific genes are downregulated within Super-enhancer Domains (Additional file 2: Figure S3H). We also examined Polycomb Domains (PDs) for evidence of dysregulation in STAG mutant cells. PDs maintain repressive chromatin states within a DNA loop, and contain genes that can increase in expression upon the loss of the DNA loop and Polycomb-mediated repression [38]. Like the SDs, there were similar numbers of DEGs identified within PDs in both *Stag1*^−*/*−^ and *Stag2*^−*/*−^ cells, but their expression levels were not significantly altered compared to all DEGs (Fig. 3f). Also, there was not a specific trend for *Stag1*^−*/*−^ specific, common, or *Stag2*^−*/*−^ specific genes being up or downregulated within PDs (Additional file 2: Figure S3H). These results suggest a specific function for STAG2, but not STAG1, in regulating gene expression at Super-enhancer Domains.

Pluripotency and differentiation genes often reside within insulated neighborhoods in mESCs. Although the pluripotency genes *Pou5f1*, *Sox2,* and *Nanog* were not significantly altered by loss of STAG1 or STAG2, the ectodermal genes *Pax6* and *Nestin* were increased and the endodermal genes *Gata6* and *Sox17* were decreased (Fig. 3g). These results suggest that whereas STAG2 may play a distinct role in regulating gene expression within SDs, both STAG proteins are required for proper maintenance of stem cell identity gene expression programs. We conclude that STAG1 and STAG2 exhibit both distinct and overlapping roles in gene expression and, ultimately, control of cellular identity.

### Dual loss of STAG1 and STAG2 reveals redundant roles in cohesin function

**Fig. 4:**
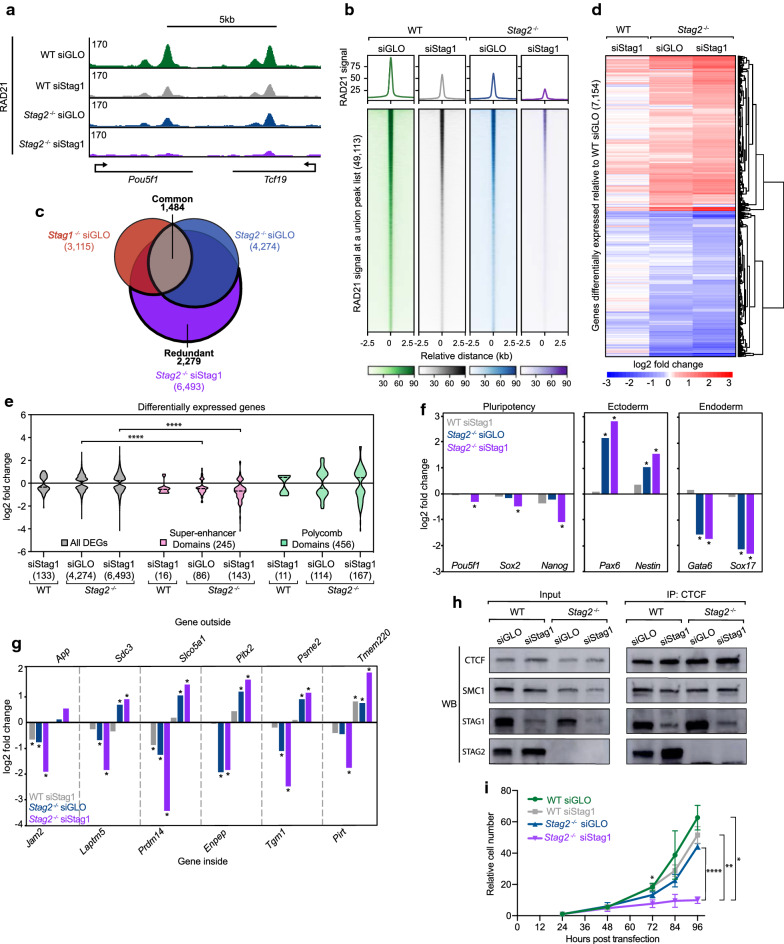
Dual loss of STAG1 and STAG2 reveals redundant functions. **a** Genome browser tracks for RAD21 ChIP-seq signal in the four conditions: wild-type siGLO, wild-type siStag1, *Stag2*^−*/*−^ siGLO, and *Stag2*^−*/*−^ siStag1 cells. **b** Average signal plots and heatmaps of RAD21 signal at a union peak list from the four conditions. **c** Venn diagram of DEGs from *Stag1*^−*/*−^ siGLO, *Stag2*^−*/*−^ siGLO, and *Stag2*^−*/*−^ siStag1 cells. **d** Clustered heatmap of log2 fold changes for a combined list of DEGs in wild-type siStag1, *Stag2*^−*/*−^ siGLO, and *Stag2*^−*/*−^ siStag1 cells all relative to wild-type siGLO. **e** Violin plots depicting log2-fold changes of all DEGs, and those within Super-enhancer Domains and Polycomb Domains for wild-type siStag1, *Stag2*^−*/*−^ siGLO, and *Stag2*^−*/*−^ siStag1 cells all relative to wild-type siGLO. Dotted lines indicate the mean. Asterisks indicate significant differences between groups (****p < 0.0001). **f** Bar graphs with log2 fold change of expression of cell identity genes including those that represent pluripotency (*Pou5f1, Sox2, Nanog*), ectoderm (*Pax6* and *Nestin*), and endoderm lineages (*Gata6* and *Sox17*) in wild-type siStag1, *Stag2*^−*/*−^ siGLO, and *Stag2*^−*/*−^ siStag1 cells, all relative to wild-type siGLO. Asterisks indicate significant differences from wild-type siGLO cells (padj < 0.01). **g** Bar graph with log2 fold change of expression of genes inside and outside of Super-enhancer Domains in wild-type siStag1, *Stag2*^−*/*−^ siGLO, or *Stag2*^−*/*−^ siStag1 cells, all relative to wild-type siGLO. Asterisks indicate significant differences from wild-type siGLO cells (padj < 0.01). **h** Western blot analysis following co-immunoprecipitation of CTCF and cohesin in the four conditions. **i** Proliferation assay with relative cell number represented as a fraction of original plating density for the four conditions. Asterisks indicate significant differences between groups (****p < 0.0001, **p < 0.01, *p < 0.05). Significance at 72 h is between wild-type siStag1 and *Stag2*^−*/*−^ siStag1 conditions

To investigate potential redundancy of STAG1 and STAG2, we generated cells nearly devoid of both STAG proteins. STAG1 was targeted for depletion with siRNA in both wild-type and *Stag2*^−*/*−^ mESC cells, alongside a non-targeting control (siGLO) treatment. Efficient STAG1 depletion was observed and quantified by western blot (Additional file 2: Figure S4A). To investigate how cohesin distribution across the genome is impacted by loss of both STAG proteins, RAD21 ChIP-seq was performed under four conditions, employing a spike-in for quantification of relative signals: wild-type siGLO, wild-type siStag1, *Stag2*^−*/*−^ siGLO, and *Stag2*^−*/*−^ siStag1. RAD21 ChIP-seq signal was strongly decreased in *Stag2*^−*/*−^ siStag1 cells compared to the other conditions (Fig. 4a, b). The loss of RAD21 signal occurred at CTCF sites, enhancers, and promoters (Additional file 2: Figure S4B). Reduced cohesin levels on the genome were also observed by western blot of chromatin bound and unbound (nuclear soluble) fractions in the four siRNA conditions (Additional file 2: Figure S4C). Differential binding analysis revealed that at a consensus list of 31,220 RAD21 sites, siStag1 treatment caused only 511 (1.6%) sites of differential RAD21 signal in wild-type cells, while siStag1 treatment in the *Stag2*^−/−^ cell line resulted in 16,878 (54.1%) sites of differential RAD21 signal (Additional file 2: Figure S4D). Importantly, these changes do not result from differences in ChIP efficiency between the samples (Additional file 2: Figure S4E).

Since dual loss of STAG proteins reduced cohesin signal on the genome, we expected substantial impacts on gene expression. RNA-seq in the four conditions revealed that siRNA depletion of STAG1 in a *Stag2*^−*/*−^ background caused 6493 genes to be differentially expressed, versus only 133 DEGs following STAG1 depletion in the wild-type background (Additional file 2: Figure S4F, Additional file 3: Table S2). Notably, the *Stag2*^−*/*−^ siStag1 DEGs were highly correlated with *Stag2*^−*/*−^ siGLO DEGs (4284) (*R*^2^ = 0.813), and weakly correlated with *Stag1*^−*/*−^ siGLO DEGs (3115) (*R*^2^ = 0.261) (Additional file 2: Figure S4G). An overlap of the *Stag2*^−*/*−^ siStag1 DEGs with *Stag1*^−*/*−^ siGLO and *Stag2*^−*/*−^ siGLO DEGs revealed a class of genes sensitive to the loss of both STAG proteins (redundant) (Fig. 4c). STAG1 and STAG2 appear to act redundantly at these 2279 genes, with a single STAG able to compensate for the loss of the other STAG protein. This is in contrast to the 1484 genes commonly affected by loss of either STAG1 or STAG2 individually. GO term analysis was performed on the redundant gene class and identified many biological processes that were also identified in *Stag2*^−*/*−^ specific gene class, and to a lesser extent the common gene class and *Stag1*^−*/*−^ specific gene class (Additional file 2: Figure S4H, Additional file 4: Table S3). Gene expression changes in *Stag2*^−*/*−^ siStag1 were of a higher magnitude than those observed upon acute depletion of STAG1 in wild-type mESCs (Fig. 4d). These higher magnitude gene expression changes occurred at SDs, with genes within SDs being significantly reduced compared to the total pool of DEGs in *Stag2*^−*/*−^ siStag1 cells (*p* < 0.0001) (Fig. 4e). We did not observe a specific role for STAG1 at SDs, as the distribution of gene expression changes in SDs was not significantly different from that of all genes for wild-type siStag1 and *Stag1*^−*/*−^ cells (Fig. 3f, Fig. 4e). Furthermore, cell identity genes showed stronger expression changes in *Stag2*^−*/*−^ siStag1 cells compared to either *Stag2*^−*/*−^ siGLO or wild-type siStag1 cells (Fig. 4f). Loss of both STAG proteins enhanced the defects in transcriptional insulation observed upon loss of either STAG1 or STAG2, with genes inside insulated neighborhoods showing decreased expression and genes outside increasing in expression (Fig. 4g).

It has been suggested that STAG proteins serve as the interface of the cohesin–CTCF interaction [35, 36]. To investigate this possibility, we performed a CTCF co-immunoprecipitation followed by western blot in the four conditions. Strikingly, we did not observe a change in the cohesin–CTCF interaction in either the single STAG depletion or *Stag2*^−*/*−^ cells depleted of STAG1 (Fig. 4h). Finally, we asked whether the reported synthetic lethality observed upon depletion of STAG1 in STAG2-null cancer cells [39, 40] also occurred in mESCs. While loss of STAG2 alone caused a slight decrease in relative cell number over time, *Stag2*^−*/*−^ siStag1 cells showed a strong proliferation defect (Fig. 4i). While the mechanism of this synthetic interaction remains unclear, it is not due to disruption of the cohesin–CTCF interface. Rather it may be due to a defect in cohesin stability on chromatin, aberrant gene expression, and/or failure to restructure chromosomes during the cell cycle.

Taken together, this work reveals overlapping and distinct roles for STAG1 and STAG2. In mESCs, cohesin-STAG1 and cohesin-STAG2 show identical patterns of localization across the genome, and their localization is not dependent on one another. Despite their high degree of genomic overlap, STAG1 and STAG2 do not display strongly overlapping roles in gene expression. Dual loss of both STAG proteins reveals a class of genes where STAG1 and STAG2 act redundantly and demonstrates the requirement for at least one STAG protein in proper cohesin function.

## Discussion

In this study, we investigated how the complete and chronic loss of STAG1 and STAG2 impacts cohesin localization and gene expression. STAG1 and STAG2 proteins localize to the same sites across the genome, likely in independent cohesin ring complexes. Upon loss of a single STAG protein, there is not redistribution of the other STAG or the core cohesin complex across the genome to new sites. Despite their shared occupancy patterns and lack of interdependency in genome occupancy, we observe that STAG1 and STAG2 have both overlapping and distinct effects on gene expression. While many genes display differential expression following loss of a single STAG protein, loss of both STAG proteins causes an increase in DEGs, as well as reduced cohesin occupancy. Finally, we demonstrate a synthetic interaction between the two STAG proteins that prevents cellular proliferation, similar to the synthetic lethality reported in cancer cell lines [39, 40]. This work suggests that STAG proteins are not specificity factors for recruiting cohesin ring complexes to distinct sites in the genome, but rather STAG proteins differentially function in gene control at common sites across the genome.

Our results demonstrate that STAG1 and STAG2 display nearly identical distributions across the genome, despite not interacting together in a stable complex. Our finding that the STAG proteins do not physically interact corroborates similar coIP and microscopy results in mESCs and human colorectal cancer lines, and is consistent with a model where cohesin functions as a single ring, rather than as a pair of rings [8, 23, 25, 27, 32, 41, 42]. Several recent reports have found that STAG1 and STAG2 localize at many shared sites, but also localize to a subset of sites exclusively [23–25, 27]. However, our analysis in mESCs does not identify a class of sites preferentially occupied by one specific cohesin-STAG complex, and agree with a recent report that shared sites reflect a population average, where some cells have cohesin-STAG1 and others have cohesin-STAG2 at an individual site [25]. We note that relative levels of STAG1 and STAG2 appear to vary across different cell types [8, 24, 25], thus the nearly equal ratio of STAG2 to STAG1 in mESCs may contribute to their overlapping genomic distributions. These results contradict a recent report on STAG localization in mESCs, which claimed that STAG2 preferentially localizes to Polycomb-marked genes through interactions with PRC1 [23]. Several factors may account for differences between present and previous studies using wild-type mESCs, including different mouse strains, the use of spike-in normalization, and differences in data analysis or interpretation. Notably, an advantage of this study is the development of stable *Stag1*^−*/*−^ and *Stag2*^−*/*−^ isogenic cell lines in order to interrogate the distribution and function of the individual STAG proteins.

We find that loss of a single STAG protein does not cause a redistribution of cohesin to new, ectopic sites. This suggests either that the STAG proteins play redundant roles in controlling cohesin localization on chromatin, or do not direct cohesin to specific sites on the genome. Our dual depletion of the STAG proteins in mESCs indicates that at least one STAG is required for stable maintenance of cohesin on chromatin. These results in cells parallel observations in *Xenopus* extracts, in which inhibition of STAG incorporation into cohesin complexes interferes with cohesin occupancy on chromatin [8]. It is also consistent with a recent report suggesting that a STAG subunit is necessary for a conformational change within the cohesin core ring, promoting its stable association with chromatin [32]. However, we do note that simultaneous depletion of STAG1 and STAG2 fails to disrupt the interaction between the core cohesin ring and CTCF, in contrast with previous findings indicating that STAG proteins are the principal interface between cohesin and CTCF [35, 36]. Notably, this coIP was performed in the presence of a nuclease, suggesting that the cohesin-CTCF interaction is independent of DNA. Our results are consistent with recent reports in mESCs and others showing that deletion of the putative STAG-interacting domain from CTCF does not disrupt the cohesin-CTCF interaction [43–45]. It is unclear, however, if the limited amount of STAG1 residing in cells after the siRNA treatment is sufficient to maintain the cohesin-CTCF interaction. Generation of cell lines with stable deletion of one STAG and acute-inducible degradation of the other would help address this, as well as allow for a more robust exploration of how STAG proteins regulate cohesin. Together, these data indicate that the individual STAG proteins may not be necessary for the interaction between CTCF and cohesin in vivo. However, the STAG proteins are required for the stability of the cohesin complex on chromatin.

Given the overlapping distribution of STAG1 and STAG2 on the genome, it is striking that the genes regulated by the two proteins only partially overlap. Gene expression can ultimately be categorized into four groups: genes that are *Stag1*^−*/*−^ specific, *Stag2*^−*/*−^ specific, sensitive to loss of either STAG1 or STAG2 (common regulation), and those only sensitive to the loss of both STAG proteins (redundant regulation). The genes within these sets are associated with a variety of biological processes, including several linked to metabolism and developmental processes, however a prominent feature distinguishing the genes affected by either STAG was not found. The redundant gene class was not enriched for specific biological roles not found in the other three gene classes. Our gene expression changes are not consistent with the previously reported specific gene expression signatures for each STAG in various cell types, with STAG1 reported to regulate RNA processing, heart and lung development, and metabolism, and STAG2 reported to regulate stem cell maintenance, hematopoietic and nervous systems, and cardiac differentiation [16, 23, 24]. While a specific role for STAG2 in regulating Polycomb Domain genes has been reported [23], we see a somewhat specific requirement for STAG2 in promoting expression of genes within Super-enhancer Domains. Surprisingly, we find that both STAG1 and STAG2 occupy promoters independent of whether those genes are specifically regulated or commonly regulated by the STAG proteins. Furthermore, the large class of redundant genes, where the presence of at least one STAG is required for proper gene expression, suggests that both cohesin variants can localize to and function at shared genomic sites, even if they do not physically interact. Together with their overlapping distributions, this suggests that STAG proteins localize to the same set of regulatory elements across the genome, yet have partially distinct effects on gene expression at those sites.

It is unclear how STAG1 and STAG2 may differentially function at common sites. One recent report suggests cohesin-STAG1 more stably associates with chromatin than cohesin-STAG2, and that cohesin-STAG1 is enriched for SMC3 acetylation, a post-translational modification associated with stable cohesin occupancy during sister chromatid cohesion [24, 27, 46]. It is possible that cohesin-STAG1 and cohesin-STAG2 display different stabilities on DNA allowing for distinct DNA loops to form or common DNA loops with distinct properties. Consistent with this, cohesin-STAG2 is reportedly involved in shorter-range contacts within TADs, while cohesin-STAG1 may mediate more long-range contacts [23–25, 27]. However, depletion of the STAG proteins in these experiments does not cause major changes to cohesin localization. It is possible that STAG1 and STAG2 differentially impact cohesin occupancy on the genome by regulating loading, unloading, or translocation of cohesin rings. Potential interactions between STAG proteins and the cohesin loader NIPBL have not been characterized, however STAG2 has been shown to co-immunoprecipitate with the cohesin unloader WAPL, while STAG1 does not [23, 28]. Cohesin is thought to be loaded at sites of active transcription and then move along chromosome arms. It is unclear if both STAG proteins are present on translocating cohesin rings, and whether they have the same dynamic properties. While this work characterizes the distinct and overlapping roles of STAG1 and STAG2, further studies are needed to investigate the properties of distinct cohesin rings, their interaction partners, and impacts on gene expression.

## Conclusions

This work utilizes stable single-knockout mouse embryonic stem cell lines to investigate the individual chronic loss of STAG1 and STAG2 in cohesin localization and gene expression. We find that while STAG1 and STAG2 show nearly identical localization patterns throughout the genome, they do not display completely overlapping roles in gene expression. This work reveals a class of genes selectively misexpressed upon loss of both STAG1 and STAG2, indicating redundant roles for the two proteins. Loss of both proteins also causes a severe depletion of cohesin on chromatin, enhanced gene expression changes, and a cell proliferation defect. This reveals that cells are dependent on the presence of at least one STAG protein for proper function. These results contribute to knowledge of cohesin’s role in genome structure and genome function during development and in disease contexts.

## Methods

### Cell culture

Murine embryonic stem cells (v6.5, male) were grown on gelatinized tissue culture dishes under standard ESC conditions [47]. HEK293T (female human embryonic kidney) cells for spike-in normalization were passaged similarly to mESCs and grown in DMEM (Gibco) supplemented with 10% cosmic calf serum, 1X GlutaMAX, 100 U/ml penicillin, and 100 μg/ml streptomycin (Thermo Fisher Scientific).

### Genome editing

mESCs were transfected with plasmids containing a sgRNA, Cas9, and a fluorescent gene (eGFP or mCherry) using Lipofectamine 2000 (Thermo Fisher Scientific). One to two days later, single fluorescent cells were either sorted by UNC Flow Cytometry Core Facility staff using a FACSAria II (BD Biosciences) or on a CytoSort Array using a CellRaft AIR System (Cell Microsystems). Cells were collected, expanded, screened by PCR and DNA sequencing, and cryogenically stored. Sequences at the editing sites were determined by PCR of the region surrounding the mutated site and Sanger sequencing. The sgRNA sequence, edited sequence, and official allele name according to the International Committee on Standardized Genetic Nomenclature for Mice are shown below.

*Stag1*^−*/*−^ replicate 1 (also known as *Stag1*^*em1Jdow*^) contains a homozygous deletion of 114 bp in exon 7 (Ile49-Thr86) and was generated with these sgRNAs:

sgRNA 1: 5′- TATATTGACACTGTCGAATC -3′

Stag1 sgRNA 2: 5′- AGGCATACAAGTACCCTTGC -3′

*Stag1*^−*/*−^ replicate 2 (also known as *Stag1*^*em2Jdow*^) contains a homozygous deletion of 121 bp in exon 7 (Ile49-Ala88) and was generated with these sgRNAs:

Stag1 sgRNA 1: 5′- TATATTGACACTGTCGAATC -3′

Stag1 sgRNA 2: 5′- AGGCATACAAGTACCCTTGC -3′

*Stag2*^−*/*−^ replicate 1 (also known as *Stag2*^*em1Jdow*^) contains a homozygous deletion of 7 bp at Leu203 and was generated with this sgRNA:

Stag2 sgRNA 1: 5′- ACTGTCATTTCACTTCTTAC -3′

*Stag2*^−*/*−^ replicate 2 (also known as *Stag2*^*em2Jdow*^) contains a homozygous deletion of 431 bp from exon 7 to 9 (Asp155-Arg298) and was generated with these sgRNAs:

Stag2 sgRNA 2: 5′- GATTACCCACTTACCATGGC -3′

Stag2 sgRNA 3: 5′- CCCACTTACCATTACAGGTA -3′

### Chromatin immunoprecipitation (ChIP) and ChIP-sequencing

Cells (3 × 10^7^) were counted and crosslinked with 1% formaldehyde in PBS for 5 min then quenched with 2.5 M glycine. Before chromatin extraction, 5% of HEK293T cells was added to the mESC samples to be later used as a spike-in normalization. Crosslinked cells were lysed with in 10 ml Lysis Buffer 1 (50 mM Hepes–KOH pH 7.5, 140 mM NaCl, 1 mM EDTA, 10% glycerol, 0.5% NP-40, and 0.25% Triton X-100) by rotating for 10 min at 4 C. After pelleting, nuclei were lysed in 5 ml Lysis Buffer 2 (10 mM Tris–HCl pH 8, 200 mM NaCl, 1 mM EDTA, and 0.5 mM EGTA) by rotating for 10 min at room temperature. After washing with 5 ml of shearing buffer (10 mM Tris pH 7.5, 1 mM EDTA, and 0.1% SDS), chromatin was resuspended in 1 ml of shearing buffer. Sonication of nuclei was performed on a Covaris E220 with the following settings: Duty Factor 5, PIP/W 140, and 200 cycles per burst for 12 min. Chromatin fragments of 200–1000 base pairs were generated. Following sonication, insoluble material was pelleted and removed by spinning samples for 10 min at 15,000 rpm.

The antibodies were incubated with 30 μl Protein G Dynabeads (Thermo Fisher Scientific) for 6–8 h prior to addition of chromatin. Unbound antibody was removed by washing beads twice with PBS with 1× protease inhibitor cocktail (PIC). The chromatin in shearing buffer was spiked with NaCl and Triton X-100 to be in a ChIP buffer (15 mM Tris pH 7.5, 1.5 mM EDTA, 0.1% SDS, 150 mM NaCl, and 1% Triton X-100). Chromatin from 1 × 10^7^ cells was added to antibody conjugated beads and incubated rotating overnight at 4 C.

The next day, beads were washed with ChIP buffer, wash buffer 1 (20 mM Tris–HCl pH 8, 500 mM NaCl, 2 mM, EDTA, 0.1%, SDS, and 1% Triton X-100), wash buffer 2 (10 mM Tris–HCl pH 8, 250 mM LiCl, 1 mM EDTA, and 1% NP-40), and wash buffer 3 (10 mM Tris pH 8, 1 mM EDTA, and 50 mM NaCl) each for 5 min rotating at 4 C. Chromatin was eluted from beads by adding elution buffer (50 mM Tris pH 8, 10 mM EDTA, and 1% SDS) and incubating at 65 C for 1 h, vortexing every 15 min. Supernatant was left at 65 C overnight with addition of 5 μl Proteinase K to reverse crosslinks. The next day, DNA was purified using a ChIP DNA Clean and Concentrate kit (Zymo). Sequencing libraries were prepared using Kapa Hyper Prep Kit (Kapa Biosystems). All sequencing was performed on three different machines. STAG1 wild-type replicate 1, STAG2 wild-type replicate 1, STAG2 *Stag1*^−*/*−^ both replicates, and STAG1 *Stag2*^−*/*−^ replicate 1, were sequenced on a NovaSeq collecting 100 bp single-end reads. STAG1 wild-type replicate 2, STAG2 wild-type replicate 2, STAG1 *Stag2*^−*/*−^ replicate 2, and all siRNA treated samples were sequenced on a NovaSeq SP collecting 50 bp paired-end reads. The RAD21 ChIPs in wild-type and STAG knockout lines were sequenced on a HiSeq 4000 collecting 50 bp single-end reads. ChIPs were performed using the antibodies referred to in Additional file 1: Table S1.

### ChIP-qPCR

STAG1 ChIP and STAG2 ChIP was performed for two biological replicates each in wild-type, *Stag1*^−*/*−^, and *Stag2*^−*/*−^ cells. Proteinase K digestion and DNA purification was performed as described above for chromatin immunoprecipitations. DNA from the ChIPs and 5% input material from each sample was analyzed by qPCR using an Applied Biosystems QuantStudio6 qPCR machine. One negative control region and three CTCF sites were examined using the following primers:

Negative control forward: 5′- GCCTAAACGGCCCACTTACT -3′

Negative control reverse: 5′- GCTCAGAGTACCCTGGAGAAT -3′

Site #1 (*Gnai2*) forward: 5′- ACAGAGCGATACGGCTCAGCAA -3′

Site #1 (*Gnai2*) reverse: 5′- AAGTGGTAGCCGAAGGCAAGTGAA -3′

Site #2 (*Jam2* SD) forward: 5′- CCCTAGTGTCTGAATGCTGAAT -3′

Site #2 (*Jam2* SD) reverse: 5′- AAGCTCTCTAAGGCTGTGTTG -3′

Site #3 (*Dmtn* SD) forward: 5′- CCTTCTGCAGACGTTCCAT -3′

Site #3 (*Dmtn* SD) reverse: 5′- ACGTCTGTCCTCTCCAAGT -3′

Average fold change of ChIP enrichment was determined relative to the negative control region and 5% input material using Microsoft Excel. Three technical replicates were performed for each biological replicate. The mean average fold change and standard deviation of the six total samples per genotype were calculated and presented as bar graphs.

### RNAi

Cells were counted and 5 × 10^5^ were plated per well in 6-well plates. 50 nM of siStag1 (Dharmacon, M-041989-01-0005) or siGLO transfection control (Dharmacon, D-001630-01-05) was transfected per well using DharmaFECT 1 (Dharmacon) transfection reagent following manufacturer’s instructions. Cells were harvested after 48 h for ChIP, protein extractions, or RNA (a timepoint prior to any cell death that occurs following incubation in siRNA reagents).

### RNA-sequencing

Three replicates of a single CRISPR clone were used for each genotype. Replicate one was used for *Stag1*^−*/*−^ and replicate two was used for *Stag2*^−*/*−^. Cells (7 × 10^5^) were plated per well into 6-well dishes and collected 2 days later. Cells were resuspended in Trizol (Invitrogen) and Chloroform (Sigma Aldrich) was added for phase separation and RNA was precipitated. After collection, RNA was purified using the Zymo RNA Clean and Concentrator Kit (Zymo). Libraries were prepared, with poly-A transcript enrichment and sequenced by Novogene on a NovaSeq 6000 instrument collecting 150 bp paired-end reads. RNA-sequencing samples are outlined in Additional file 1: Table S1.

### Co-immunoprecipitation (coIP)

Co-immunoprecipitation assays were performed using a Nuclear Complex Co-IP Kit (Active Motif) with a homemade protocol for the nuclear fraction digestion step. Cells were collected via scraping in PBS from a confluent tissue culture plate. Nuclei were isolated using the kit procedure. Nuclei were lysed in 200 μl Buffer A (10 mM HEPES pH 7.9, 10 mM KCl, 1.5 mM MgCl_2_, 340 mM Sucrose, and 10% glycerol) with 1× protease inhibitor cocktail (PIC) (Sigma Aldrich) and digested with 10U of Benzonase at 37 C for 15 min. The reaction was quenched with 0.5 M EDTA and incubated on ice for 5 min. Supernatant containing chromatin-bound proteins was collected following centrifugation at 4 C for 5 min at 5000×*g*. Protein levels were quantified using the DC Protein Assay (BioRad). 400 μg of protein was used in each IP following the kit procedure, using the low stringency buffer. Protein G Dynabeads were incubated with antibody for 6–8 h and used for capturing and cleaning IP material following kit instructions (Invitrogen). IP material was eluted in 50 μl of ChIP Elution Buffer (50 mM Tris pH 8, 10 mM EDTA, and 1% SDS) at 65 C for 1 h, vortexing every 15 min to keep beads in suspension.

### Fractionation

Cells were trypsinized and counted 48 h post-transfection of siRNA in order to obtain 1 x 10^7^ cells per condition. Chromatin bound and unbound (nuclear soluble) fractions were collected using the Subcellular Protein Fractionation Kit for Cultured Cells (Thermo Scientific). Collection of fractions was performed following manufacturer’s instructions for the 100 μl packed cell volume, with additional PBS washes in between each collection.

### Western blotting

Adherent cells of similar confluency were washed with PBS and collected via scraping. Cell pellets were frozen on dry ice and stored until use. Once thawed, pellets were resuspended in 10 ml of Lysis Buffer A (10 mM HEPES pH 7.9, 10 mM KCl, 0.1 mM EDTA, and 0.1 mM EGTA) containing 1× protease inhibitor cocktail (PIC) (Sigma Aldrich) and incubated at 4 C while rocking for 15 min. 1 ml of 10% NP-40 was added, samples immediately vortexed, and pelleted at 1350×*g* for 5 min at 4 C. The pellet was resuspended in 1 ml of cold Buffer TEN250/0.1 (50 mM Tris–HCl pH 7.5, 250 mM NaCl, 5 mM EDTA, and 0.1 mM NP-40) containing 1X PIC and incubated for a minimum of 30 min rotating at 4 C. After spinning at 4 C at max speed for 10 min, the nuclear fraction (supernatant) was collected.

Protein levels were quantified using the DC Protein Assay (BioRad). Samples were run on 4–20% Tris–Glycine gels (BioRad) and transferred to nitrocellulose membranes (VWR). Membranes were blocked for 1 h with 5% blocking grade buffer (BioRad) and incubated overnight rocking at 4 C with primary antibody. Antibodies used were SMC1 (Bethyl, A300-055A), SMC3 (Abcam, ab9263), RAD21 (Bethyl, A300-080A), STAG1 (Bethyl, A300-157A), STAG2 (Bethyl, A300-158A), CTCF (Active Motif, 31917004), Histone H3 (Abcam, ab1791), and Actin (Abcam, ab190476). Membranes were washed 3 × 10 min with TBS-T at room temperature and incubated for 1 h rocking at 4 C with secondary antibody. Antibodies used were Donkey anti-Rabbit (GE Healthcare, NA934) and Rabbit anti-Goat (Abcam, ab97100). Secondary antibody was washed off with 3 × 10 min washes with TBS-T at room temperature. Membranes were imaged using either Thermo SuperSignal West Pico PLUS or Thermo SuperSignal West Femto chemiluminescent substrates with an Amersham Imager 600 (GE Healthcare). Quantification of blots was performed using ImageQuant TL (GE Healthcare).

### Proliferation

Cells were trypsinized and counted 24 h post-transfection of siRNA. Cells (5 × 10^4^) were plated in single well of a 6-well plate for every timepoint. Total cell count per well was counted using a Countess II FL (Life Technologies) at 48 h, 72 h, 84 h, and 96 h post-transfection. All calculations are represented as a fraction of initial plating density and plotted using GraphPad Prism (GraphPad). Significance was determined using Tukey’s multiple comparisons test.

### ChIP-seq analysis, normalization, and representations

Biological replicates were merged as raw fastq files before being processed. Merged files were then aligned to a merged genome containing both mouse genome assembly mm10 and human genome assembly hg38 using bowtie (v 1.2.2) (parameters -v 2 -p 24 -S -m 1 -best -strata). Mouse chromosomes were denoted by a Mchr prefix for future distinction from human chromosomes. Duplicate sequences were removed using samtools (v 1.9) markdup (-r -s). Reads mapping to mouse and human chromosomes were separated using samtools idxstats and counted with awk. A bam file containing only mouse reads was created using samtools view. This was then converted to bed format using bedtools (v 2.26) bamtobed and reads were extended by 200 bp. Extended bed files were used to call peaks using MACS (v 2016-02-15) with a false discovery rate of 1% (macs2 callpeak -f BED -g mm -q 0.01). To obtain a high confidence peak set prior to any peak related analysis, peak summits were expanded by 50 bp on either side and any expanded peaks overlapping a repeat element (defined using the Repeat Masker Track from UCSC genome browser) were removed using bedtools intersect (-v).

A normalization factor was calculated for each ChIP-seq dataset using the formula 1/h where h is the number of human aligned reads in millions as described previously [48]. The bed file containing mouse reads was converted to a bedgraph file using bedtools genomecov (-bga -scale 1/h) before being converted to a bigwig file with bedGraphToBigWig from ucsctools (v 320). *Z*-score normalization was performed where indicated using a custom R script from Spencer Nystrom of Dr. Daniel McKay’s lab.

Summit files generated by MACS were extended by 50 bp on either side before performing peak overlaps with bedtools intersect. Average signal plots were generated using deeptools (v 3.0.1) computeMatrix (reference-point for CTCF sites, promoters, enhancers, and denoted peak lists) followed by deeptools plotProfile. Heatmaps were generated using deeptools computeMatrix (reference-point) followed by deeptools plotHeatmap. Clustering of heatmaps was performed with k-means clustering with 3 clusters using deeptools plotHeatmap (-kmeans 3). Fingerprint plot was generated using deeptools plotFingerprint (-skipZeros). Correlation plots (Additional file 2: Figure S2) were generated using deeptools multiBigwigSummary followed by plotCorrelation (-removeOutliers -skipZeros -corMethod pearson). Signal tracks were visualized using IGV 2.4.10 desktop browser. Differentially bound sites were identified using DiffBind (v 2.12.0) [34].

The list of promoters was obtained from UCSC transcription start sites. Enhancers were defined as sites co-occupied by the transcription factors OCT4, SOX2, and NANOG. ChIP-seq data for these factors was pooled and peaks were called following the method above. Any transcription start site bound by these factors was removed from the list of enhancers. “Other” sites were obtained by taking the list of peaks for each class and removing those that overlapped with CTCF sites, enhancers, and promoters using bedtools intersect (-v).

### RNA-seq analysis

RNA sequencing reads were aligned to the genome using Star (version 2.6.0a) [49]. Differentially expressed genes were identified using DESeq 2 from Bioconductor (v 1.24.0) [50]. Lists of DEGs for all the overlaps and combined lists of DEGs were generated in R using dplyr. The correlation plot was generated using GraphPad PRISM followed by Pearson correlation analysis. Heatmaps of log2 fold changes were generated in R using pheatmap. For gene ontology (GO) analysis, the lists of *Stag1*^−*/*−^ specific, *Stag2*^−*/*−^ specific, common, and redundant differentially expressed genes were further subsetted into upregulated and downregulated gene sets. GO analysis was performed on each of these six gene sets using the ShinyGO software package (FDR < 0.05) [51]. The top 30 significantly enriched terms for each subset was then intersected with the others. FDRs for the terms with at least one overlap in another gene set were represented as a heatmap using pheatmap in R. The top 30 enriched terms for each subset are presented in Additional file 4: Table S3, with the exception of the *Stag1*^−*/*−^ specific downregulated terms only having 23. Gene expression counts from 1019 transformed human cell lines were downloaded from the Cancer Cell Line Encyclopedia. Expression of STAG proteins was represented as a ratio of Stag2 to Stag1 transcript levels, and plotted as a cumulative distribution function using Microsoft Excel. Read counts from Stag1 and Stag2 transcripts for mESC v6.5 were determined from our own RNA-seq data. Violin plots and statistics were generated using GraphPad PRISM followed by a Wilcoxon ranked-sum test. MA plots were generated using DESeq 2 plotMA. Coordinates of insulated neighborhoods (Super-enhancer Domains and Polycomb Domains) were obtained from [38]. The original coordinates were converted from mm9 to mm10 using UCSC LiftOver. Bar graphs of cell identity genes, genes exhibiting the loss of insulation signature, transcript ratios, and percentage of genes changed in SD and PDs were manually generated with Microsoft Excel. Genes located within or near the insulated neighborhoods were identified using bedtools intersect.

## Supplementary information

**Additional file 1: Table S1.** Accession numbers of data used in this study. Summary table of the datasets used in this study including antibody product number, genotypes, factor accession numbers, as well as background accession numbers.

**Additional file 2.** Additional figures and corresponding legends for each complimentary main text figure (S1, S2, S3, and S4).

**Additional file 3: Table S2.** Differentially expressed genes for all depletion conditions compared to wild-type siGLO. Full lists of differentially expressed genes for the four conditions used in RNA-seq analysis in this study, all compared to wild-type siGLO (*Stag1*^−*/*−^ siGLO, wild-type siStag1, *Stag2*^−*/*−^ siGLO, and *Stag2*^−*/*−^ siStag1).

**Additional file 4: Table S3.** Gene ontology analysis of differentially expressed genes from *Stag1*^−*/*−^*, Stag2*^−*/*−^, or *Stag2*^−*/*−^ siStag1 mESCs. Lists of the top 30 GO-terms for both up- and down-regulated genes in the four gene classes (common*, Stag1*^−*/*−^ specific, *Stag2*^−*/*−^ specific, and redundant).

## Data Availability

The datasets supporting the conclusions of this article are available in the GEO repository under accession number GSE144116 and can be accessed by going to https://www.ncbi.nlm.nih.gov/geo/query/acc.cgi?acc=GSE144116. All individual accession numbers are available in Additional file 1: Table S1.

## Abbreviations

mESC: Mouse embryonic stem cell
ChIP-seq: Chromatin immunoprecipitation followed by sequencing
coIP: Co-immunoprecipitation
IP: Immunoprecipitation
RNA-seq: RNA sequencing
DEGs: Differentially expressed genes
SD: Super-enhancer Domain
PD: Polycomb Domain
FDR: False discovery rate
LFC: Log2-fold change
GO: Gene ontology
