# Supplementary material for: Distinct and overlapping roles of STAG1 and STAG2 in cohesin localization and gene expression in embryonic stem cells
Source: Epigenetics Chromatin. 2020 Aug 10;13:32. doi: 10.1186/s13072-020-00353-9 (PMC7418333; doi:10.1186/s13072-020-00353-9)
Supplement: Supplementary file 1 — Additional file 1: Table S1. Accession numbers of data used in this study. Summary table of the datasets used in this study including antibody product number, genotypes, factor accession numbers, as well as background accession numbers. [file 13072_2020_353_MOESM1_ESM.pdf]

Table S1. Accession numbers of data used in this study

Data Type	Factor	Antibody	Cell Type	Factor Accession Number	Background Accession Number
ChIP-seq	STAG1	Bethyl A300-0157A	WT mESC	GSM4280480<br>GSM4280481	GSE144116
	STAG2	Bethyl A300-0159A	WT mESC	GSM4280482<br>GSM4280483	GSE144116
	RAD21	Bethyl A300-080A	WT mESC	GSM4074281<br>GSM4074282	GSE137285
	CTCF	Active Motif 61311	WT mESC	GSM4074279<br>GSM4074280	GSE137285
	OCT4	Santa Cruz sc-8628X	WT mESC	GSM1082340	GSE44286
	SOX2	R&D Systems MAB2018	WT mESC	GSM1082341	GSE44286
	NANOG	Bethyl A300-397A	WT mESC	GSM1082342	GSE44286
	RAD21	Abcam ab992	WT mESC	GSM4280484<br>GSM4280485	GSE144116
	STAG1	Bethyl A300-0157A	*Stag2* $^{-/-}$ mESC	GSM4280486<br>GSM4280487	GSE144116
	RAD21	Abcam ab992	*Stag2* $^{-/-}$ mESC	GSM4280488<br>GSM4280489	GSE144116
	STAG2	Bethyl A300-0159A	*Stag1* $^{-/-}$ mESC	GSM4280490<br>GSM4280491	GSE144116
	RAD21	Abcam ab992	*Stag1* $^{-/-}$ mESC	GSM4280492<br>GSM4280493	GSE144116
	RAD21	Bethyl A300-080A	WT siGLO mESC	GSM4280494<br>GSM4280495	GSE144116
			WT siStag1 mESC	GSM4280496<br>GSM4280497	GSE144116
			Stag2 $^{-/-}$ siGLO mESC	GSM4280498<br>GSM4280499	GSE144116
			Stag2 $^{-/-}$ siStag1 mESC	GSM4280500<br>GSM4280501	GSE144116
RNA-seq	-	-	WT siGLO mESC	GSM4280519<br>GSM4280520<br>GSM4280521	GSE144116
	-	-	*Stag1* $^{-/-}$ siGLO mESC	GSM4280522<br>GSM4280523<br>GSM4280524	GSE144116
	-	-	WT siStag1 mESC	GSM4280525<br>GSM4280526<br>GSM4280527	GSE144116
	-	-	*Stag2* $^{-/-}$ siGLO mESC	GSM4280528<br>GSM4280529<br>GSM4280530	GSE144116
	-	-	*Stag2* $^{-/-}$ siStag1 mESC	GSM4280531<br>GSM4280532<br>GSM4280533	GSE144116
