# Supplementary material for: Distinct and overlapping roles of STAG1 and STAG2 in cohesin localization and gene expression in embryonic stem cells
Source: Epigenetics Chromatin. 2020 Aug 10;13:32. doi: 10.1186/s13072-020-00353-9 (PMC7418333; doi:10.1186/s13072-020-00353-9)
Supplement: Supplementary file 2 — Additional file 2. Additional figures and corresponding legends for each complimentary main text figure (S1, S2, S3, and S4). [file 13072_2020_353_MOESM2_ESM.pdf]

Figure S1. STAG1 and STAG2 occupy the same sites across the genome

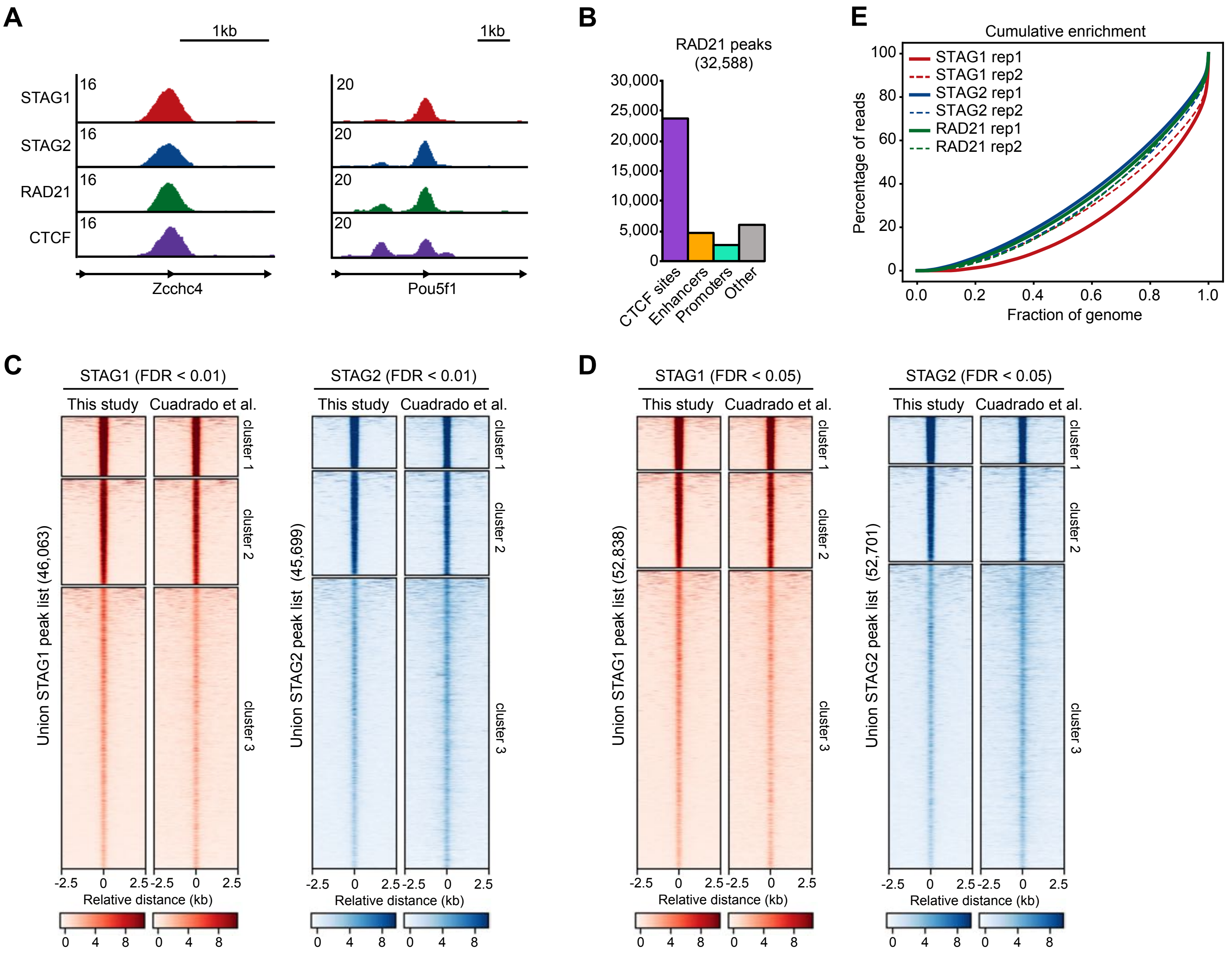

Supplemental Figure 1. STAG1 and STAG2 occupy the same sites across the genome

A. Genome browser tracks for STAG1, STAG2, RAD21, and CTCF at example loci (*Z*-score normalized).

B. Frequency of RAD21 peaks overlapping known functional elements in the genome: CTCF sites, enhancers, promoters, or other (none of the above).

C. Heatmaps of STAG1 and STAG2 ChIP-seq signal at union peak lists from peaks called in this study and peaks re-analyzed from Cuadrado et al., 2019, using FDR < 0.01. Heatmaps are clustered using k-means and ChIP-seq signal is *Z*-score normalized.

D. Heatmaps of STAG1 and STAG2 ChIP-seq signal at union peak lists from peaks called in this study and peaks re-analyzed from Cuadrado et al., 2019, using FDR < 0.05. Heatmaps are clustered using k-means and ChIP-seq signal is *Z*-score normalized.

E. Fingerprint plot showing cumulative enrichment of reads per fraction of the genome for each ChIP-seq sample consisting of two biological replicates.

Figure S2. Cohesin localization is largely independent of either STAG protein

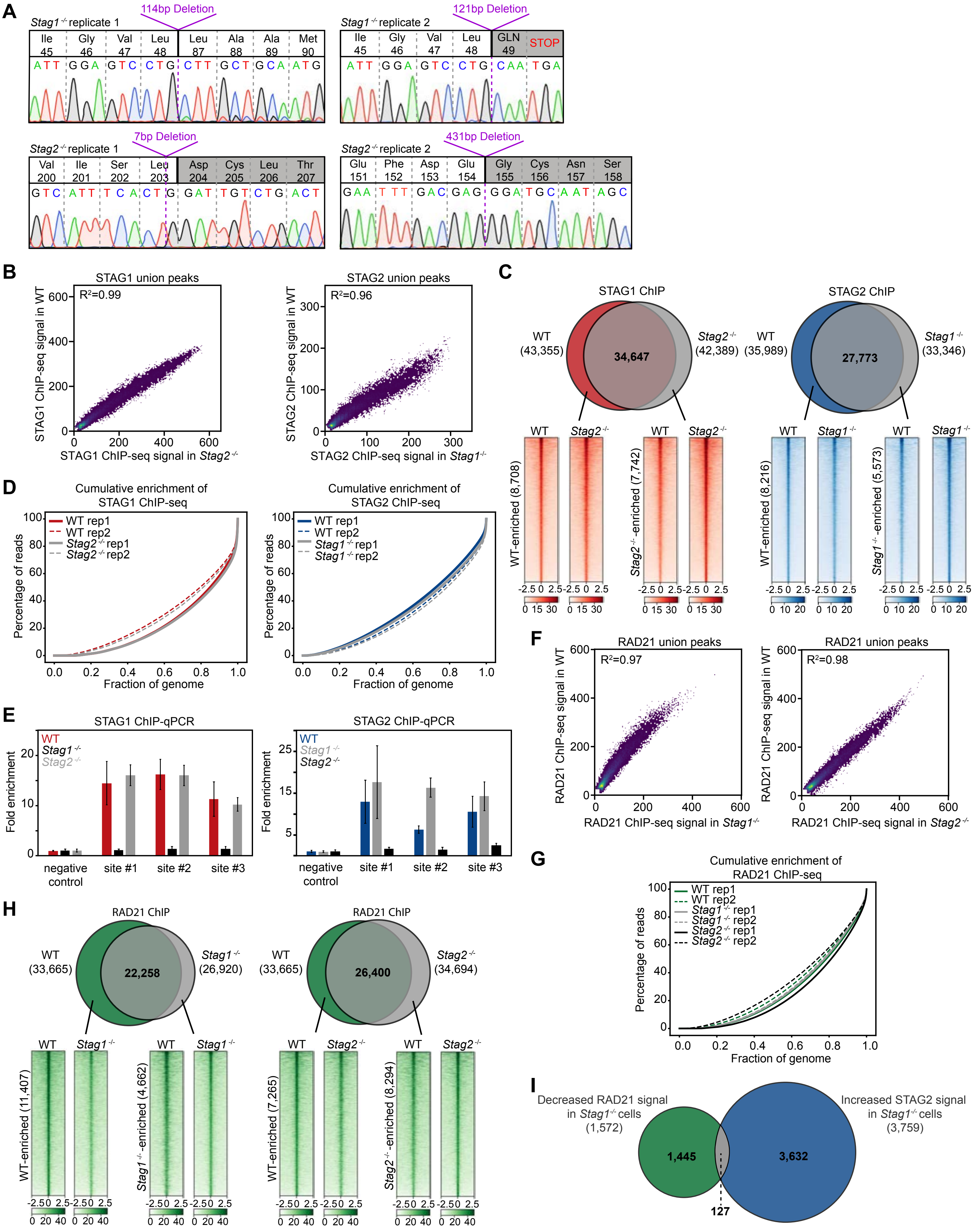

Supplemental Figure 2. Cohesin localization is largely independent of either STAG protein

A. Sequencing chromatograms for *Stag1* $^{-/-}$ and *Stag2* $^{-/-}$ mESC lines. Gray highlight indicates new amino acid sequence and purple dashed lines indicate the specific cut sites.
B. Correlation plots of STAG1 signal at a set of union peaks from wildtype and *Stag2* $^{-/-}$ cells and STAG2 signal at a set of union peaks from wildtype and *Stag1* $^{-/-}$ cells.
C. Venn diagram of STAG1 peak overlap between wildtype and *Stag2* $^{-/-}$ cells. Heatmaps depicting signal at wildtype-enriched or knockout-enriched sites are also shown. The same is shown for STAG2 peak overlap in wildtype and *Stag1* $^{-/-}$ cells.
D. Fingerprint plot showing cumulative enrichment of reads per fraction of the genome for STAG1 ChIP-seq replicates in wildtype and *Stag2* $^{-/-}$ cells. STAG2 ChIP-seq replicates in wildtype and *Stag1* $^{-/-}$ cells is also shown.
E. ChIP-qPCR for STAG1 and STAG2 in wildtype, *Stag1* $^{-/-}$, and *Stag2* $^{-/-}$ cells. Fold enrichment at three different CTCF sites, relative to 5% input material and a negative control region is depicted. Data represented as mean ± standard deviation across two biological replicates, each with three technical replicates.
F. Correlation plots of RAD21 signal at a set of union peaks from wildtype and *Stag1* $^{-/-}$*,* and wildtype and *Stag2* $^{-/-}$ cells.
G. Fingerprint plot showing cumulative enrichment of reads per fraction of the genome for RAD21 ChIP-seq in the two biological replicates for wildtype, *Stag1* $^{-/-}$, and *Stag2* $^{-/-}$ cells.
H. Venn diagram of RAD21 peak overlap between wildtype and *Stag1* $^{-/-}$ cells, and wildtype and *Stag2* $^{-/-}$ cells. Heatmaps depicting signal at wildtype-enriched or knockout-enriched sites are also shown.
I. Venn diagram of the overlap between sites of decreased RAD21 ChIP signal in *Stag1* $^{-/-}$ cells compared to wildtype, and sites of increased STAG2 ChIP signal in *Stag1* $^{-/-}$ cells compared to wildtype.

Figure S3. Roles of STAG1 and STAG2 in gene expression

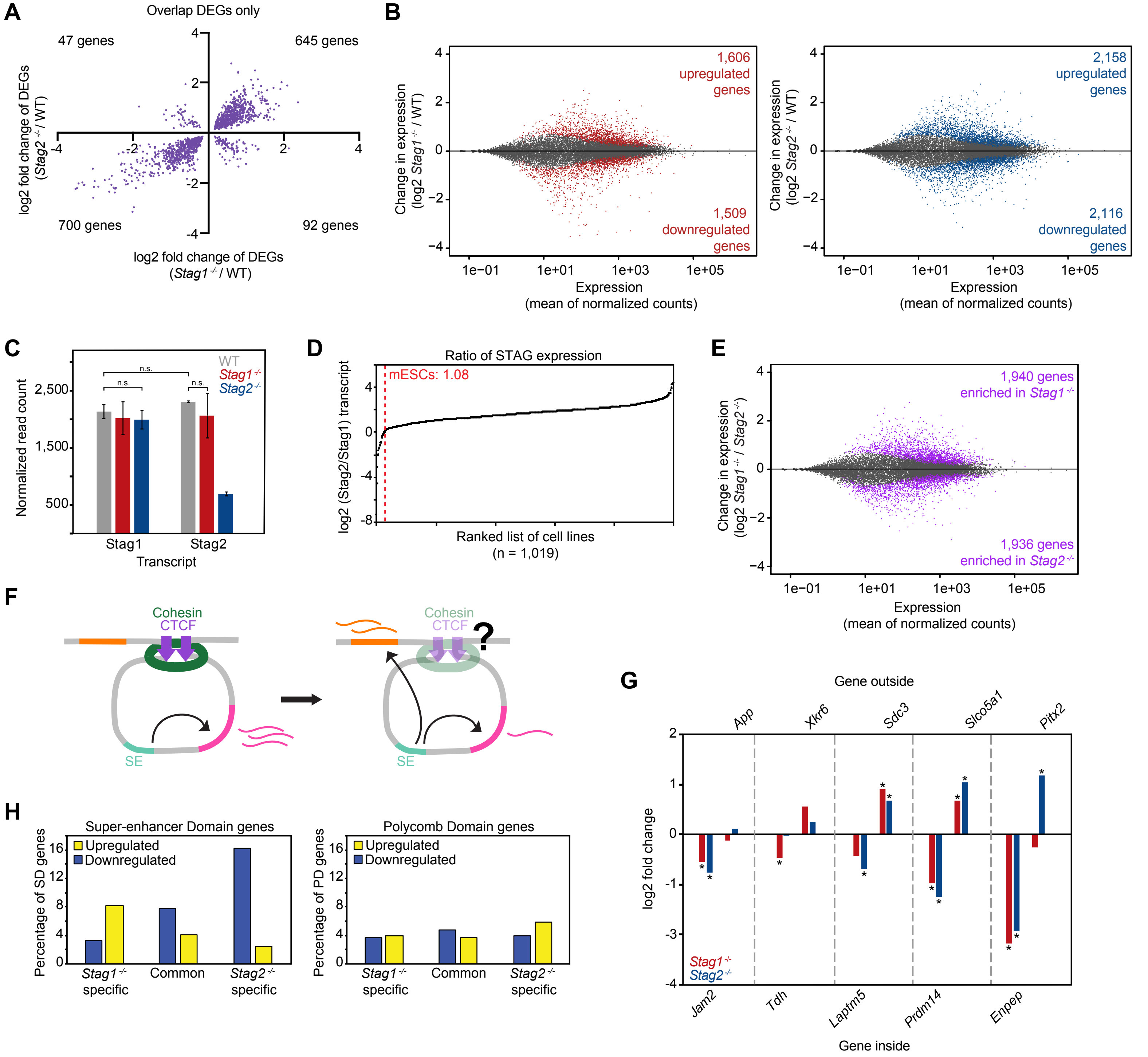

Supplemental Figure 3. Roles of STAG1 and STAG2 in gene expression

A. Correlation plot showing the log2 fold changes of DEGs that are identified in both *Stag1* $^{-/-}$ and *Stag2* $^{-/-}$ cells (common only) as well as the number of genes within each quadrant of the graph.
B. MA plots showing DEGs in *Stag1* $^{-/-}$ or *Stag2* $^{-/-}$ cells relative to wildtype.
C. Normalized read counts for STAG1 and STAG2 transcripts for wildtype, *Stag1* $^{-/-}$, and *Stag2* $^{-/-}$ cells. Data represented as the average across three biological replicates with error bars representing standard deviation. The statistical test used to determine no significance (n.s) was a T-test.
D. Relative transcript levels (log2 Stag2/Stag1) in our mESCs and 1,019 cell lines pulled from the Cancer Cell Line Encyclopedia.
E. MA plot showing genes differentially expressed between *Stag1* $^{-/-}$ and *Stag2* $^{-/-}$ cells (without respect to wildtype cells).
F. Model of a Super-enhancer Domain where a cohesin and CTCF-mediated DNA loop focuses the activity of a Super-enhancer (teal) on a target gene inside the loop (pink). This prevents the Super-enhancer from acting on the gene outside the loop (orange). When transcriptional insulation is lost or impaired, the Super-enhancer can act on the orange gene and decrease activity on the pink gene.
G. Bar graph of log2 fold change of expression of genes inside and outside of Super-enhancer Domains in *Stag1* $^{-/-}$ or *Stag2* $^{-/-}$ cells relative to wildtype.
H. Bar graph of percentages of all Super-enhancer Domain genes and Polycomb Domain genes that are up and downregulated in *Stag1* $^{-/-}$ specific, common, and *Stag2* $^{-/-}$ specific gene classes.

Figure S4. Dual loss of STAG1 and STAG2 causes major changes in gene expression and cohesin localization

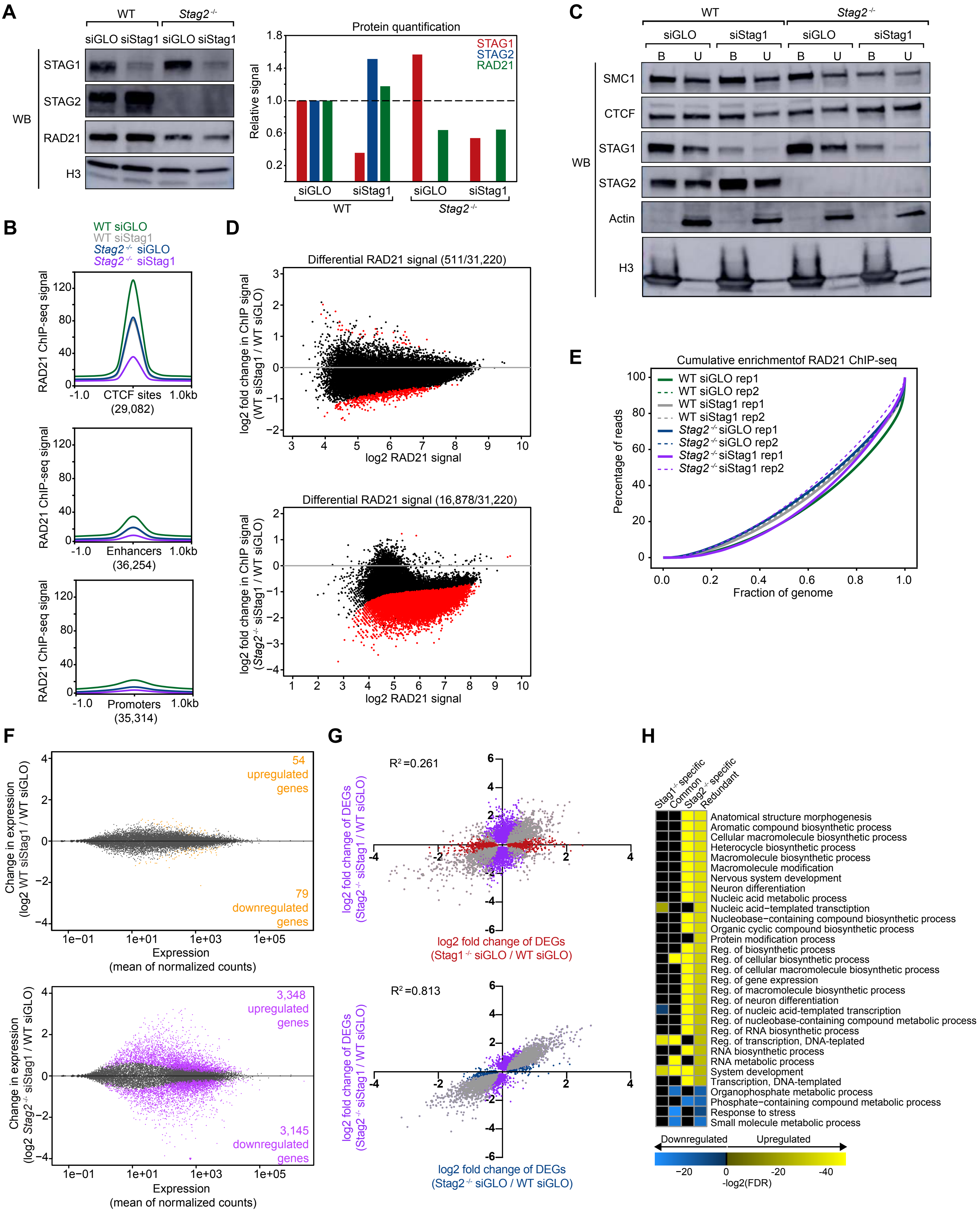

Supplemental Figure 4. Dual loss of STAG1 and STAG2 causes major changes in gene expression and cohesin localization

A. Western blot analysis and quantification of the four siRNA conditions: wildtype siGLO, wildtype siStag1, *Stag2* $^{-/-}$ siGLO, and *Stag2* $^{-/-}$ siStag1 cells.
B. Average signal plots showing RAD21 signal in the four conditions (wildtype siGLO, wildtype siStag1, *Stag2* $^{-/-}$ siGLO, and *Stag2* $^{-/-}$ siStag1 cells) at CTCF sites, enhancers, and promoters.
C. Western blot analysis following a fractionation in the four siRNA conditions. Both chromatin bound (B) and unbound, or nuclear soluble, (U) are shown for each condition.
D. MA plots showing sites of differential enrichment of RAD21 in the wildtype siStag1 and *Stag2* $^{-/-}$ siStag1 cells relative to wildtype siGLO.
E. Fingerprint plot showing cumulative enrichment of reads per fraction of the genome for RAD21 ChIP-seq in the two biological replicates for wildtype siGLO, wildtype siStag1, *Stag2* $^{-/-}$ siGLO, and *Stag2* $^{-/-}$ siStag1 conditions.
F. MA plots showing DEGs following treatment of siStag1 in wildtype and *Stag2* $^{-/-}$ cells compared to wildtype siGLO.
G. Correlation plots of log2 fold changes of DEGs between the single knockouts and dual depletion condition. *Stag1* $^{-/-}$ siGLO specific genes are in red, *Stag2* $^{-/-}$ siGLO specific genes are in blue, *Stag2* $^{-/-}$ siStag1 specific genes are in purple, and the genes that overlap within each plot are in gray.
H. Gene Ontology (GO) terms for biological processes that are *Stag1* $^{-/-}$ specific, *Stag2* $^{-/-}$ specific, common to both single knockouts, and redundant (*Stag2* $^{-/-}$ siStag1 specific).
